# The Utilisation of Computed Tomography Pulmonary Angiography in a Regional Victorian Emergency Department

**DOI:** 10.7759/cureus.40833

**Published:** 2023-06-22

**Authors:** Ali Uthuman, Tae H Kim, Subatharshini Sountharalingam

**Affiliations:** 1 Rural Health, University of Melbourne, Shepparton, AUS; 2 General Medicine, Goulburn Valley Health, Shepparton, AUS

**Keywords:** computed tomography pulmonary angiography, venous thromboembolism, emergency department, pulmonary embolism, ctpa

## Abstract

Background: Pulmonary embolism (PE) is a critical condition with various recognized risk factors. This study investigates these factors in a regional Australian population.

Aims: The primary aim is to examine the significance of traditional risk factors in the clinical decision to request a computed tomography pulmonary angiography (CTPA) scan for suspected PE within this population and assess the association between the timing of CTPA requests (office vs. after-hours) and PE occurrence.

Methods: In this single-center retrospective study, we analyzed data from 434 patients undergoing CTPA at Goulburn Valley Health's (GVH) emergency department (ED) between January and August 2022. Covariates included age, clinical indications, and medical background. Statistical tests were applied with a p-value <0.05 indicating significance.

Results: Pulmonary embolism was diagnosed in 39 (20.9%) males and 17 (6.9%) females, with a mean age of 65.04 years (SD: 16.11). Univariate regression indicated a positive association between age and PE. Multivariate analysis showed a significant positive association for unilateral lower limb (LL) swelling/deep vein thrombosis (DVT) (OR: 5.474, p=0.003) and a significant negative association for being female (OR: 0.308, p<0.001). Variables such as shortness of breath, tachycardia, syncope, and chest pain were not significantly associated with PE. No association was found between CTPA request time and PE (χ²=0.9535, df=1, p=0.3288).

Conclusion: Increasing age and unilateral LL swelling/DVT are significantly associated with PE. Some signs and symptoms showed negative or positive odds but were not statistically significant. The timing of CTPA requests did not correlate with PE incidence.

## Introduction

A pulmonary embolism (PE) is the blockage of the pulmonary artery or its branches caused by substances such as blood clots, tumors, air, or fat that have traveled from other parts of the body. This paper discusses PE resulting from blood clots.

Pulmonary embolism can present with a wide range of symptoms, from none to severe or even fatal outcomes. Common symptoms include shortness of breath (SOB), pleuritic chest pain, and signs of deep vein thrombosis (DVT) [1]. Hemoptysis is rare, while severe cases may involve shock, arrhythmia, or syncope. Some patients with large PE may show no or mild symptoms, making it crucial to maintain a high level of suspicion to avoid missing clinically significant cases.

Diagnosing PE involves clinical assessment, risk stratification, and diagnostic tests. Medical history, physical examination, and blood tests like the D-dimer assay help assess risk. Imaging techniques, such as computed tomography pulmonary angiography (CTPA) or ventilation-perfusion (V/Q) scans, are used to identify clots.

This study aims to evaluate the applicability of established risk factors for PE in a regional Australian population. Furthermore, it investigates the potential link between the timing of CTPA requests (office hours vs. after-hours) and the incidence of PE in this population.

## Materials and methods

Ethical approval and design

Approval from the Human Research Ethics Committee (HREC) of Goulburn Valley Health (GVH) was obtained (approval no. GVH 41/22) for this single-center retrospective study conducted at the GVH emergency department (ED), Shepparton, AU. To ensure the validity of the regression analysis, multicollinearity was assessed using the variance inflation factor (VIF) and tolerance statistics. No multicollinearity was found, as all VIF values were below 10 and all tolerance values were above 0.1.

Inclusion criteria

Data were collected retrospectively from all patients who underwent CTPA at the GVH ED from January to August 2022. Coding results identified patients who underwent CTPA at the ED from January to August 2022. The CTPA request forms were interrogated to identify the time of the request, the presence of the Wells' score or D-dimer, the indication for CTPA, and relevant background medical conditions. The CTPA reports interpreted by radiologists were used to confirm the diagnosis of PE and record alternate radiological diagnoses if negative for PE.

Covariates and definitions

Age was recorded as a continuous variable. Nominal covariates for an indication of CTPA included SOB, chest pain, pleuritic chest pain, tachycardia, hypoxia, hemoptysis, unilateral lower limb (LL) swelling/DVT, syncope, age, female, and presence of D-dimer (non-aged-adjusted). 

Nominal covariates for relevant background included procoagulant condition, previous DVT/PE, malignancy, and immobilization/long travel. To assess hypoxia and tachycardia, we relied on the clinicians' documentation of them on the CTPA request forms rather than conducting an individual review of each patient's medical record. For the logistic regression analysis, the variables unilateral LL swelling and current DVT, prior instances of DVT and PE, as well as recent immobilization and prolonged travel, were combined to better assess their collective impact on the outcome.

In the context of this study, we defined two distinct periods: office hours and after-hours. Office hours were designated as the time between 8:00 a.m. and 8:00 p.m., while after-hours referred to the period between 8:00 p.m. and 8:00 a.m. the following day.

Data processing and statistical analysis

Data were analyzed using GraphPad Prism (GraphPad Software Inc., San Diego, CA, USA) and SPSS Statistics (IBM Corp., Armonk, NY, USA). Nominal data significance was assessed with univariate and multivariate logistic regression tests. Additionally, the Mann-Whitney U test was employed to analyze age distribution, while the chi-square test was utilized to examine the relationship between PE occurrence, CTPA request time, and high D-dimer levels. A p-value <0.05 indicated statistical significance. Pulmonary embolism and other radiological diagnoses were calculated and reported.

## Results

In this study, 434 patient records consisting of 247 females and 187 males, were analyzed. The study diagnosed 56 (12.9%) patients with PE, with 39 of them being males (20.9% of males) and 17 females (6.9% of females). The ages of subjects diagnosed with PE ranged from 16 to 88, with a mean age of 65.04 years (SD: 16.11).

Most scan requests (46.5%) occurred between 2:00 p.m. and 6:00 p.m. The chi-square test revealed no significant association between the time of the CTPA request and the presence of PE (χ²=0.9535, df=1, p=0.3288).

Among scanned patients, 164 (37.8%) had a high D-dimer, 267 (60.8%) had no D-dimer, and one had a negative test with the scan revealing no PE. The chi-square test demonstrated no significant association between high D-dimer levels and the presence of PE (χ²=0.2817, df=1, p=0.5956). Out of 434 requests, only 11 included actual Wells' scores. Summary statistics are shown in Table 1.

**Table 1:** Summary statistics for the patient cohort PE: Pulmonary embolism

Variables	Total	PE	No PE	% PE
Patients	434	56	378	12.9
Gender				
Male	187	39	148	20.9
Female	247	17	230	6.9
D-dimer				
Positive	164	19	145	11.6
None	267	37	230	13.9
Negative	1	0	1	0.0
Time of request				
Office hours	282	33	249	11.7
After-hours	152	23	129	15.1

**Figure 1:**
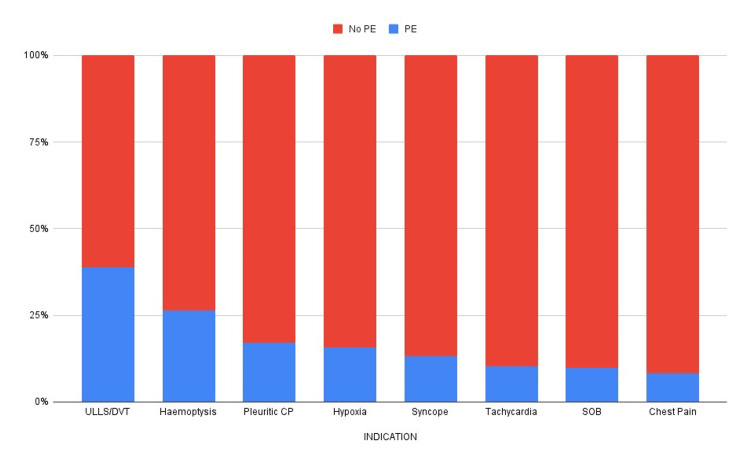
Proportion of PE cases by clinical signs and symptoms PE: Pulmonary embolism, ULLS: Unilateral lower limb swelling, DVT: Deep vein thrombosis, CP: Chest pain, SOB: Shortness of breath

The indications for CTPA were SOB (46%), chest pain (39.4%), tachycardia (17.4%), pleuritic chest pain (16.4%), hypoxia (13.1%), hemoptysis (4.4%), and unilateral LL swelling/DVT (4.14%), as seen in Figure 1.

Prothrombotic risk factors, prevalence, and PE incidence rates (in parenthesis) include procoagulant conditions (1.38%, 33.3%), malignancy (7.14%, 19.3%), previous PE/DVT (3.68%, 25%), and immobilization/long travel (3.22%, 35.7%). Among 378 non-PE CTPA reports, there were no alternative diagnoses in 70.9%. When there was an alternative diagnosis, it was consolidation (13.8%), emphysema (6.6%), atelectasis (5.3%), pleural effusion (2.9%), and lung nodules (2.6%).

The Mann-Whitney U test revealed a statistically significant difference in age distribution between patients with and without PE (U=8068, p=0.0039, two-tailed). Using univariate logistic regression (Table 2), the study found age to be significantly associated with PE diagnosis (OR: 1.024, p=0.006) and chest pain linked to lower PE incidence (OR: 0.464; p=0.018). No significant associations were observed for SOB, tachycardia, or syncope.

**Table 2:** Univariate logistic regression analysis of PE risk factors * p<0.05 CTPA: Computed tomography pulmonary angiography, PE: Pulmonary embolism, OR: Odds ratio, SOB: Shortness of breath, SD: Standard deviation

Variables	CTPA +	CTPA -	OR	p-value
SOB (number)	21	192	0.75	0.319
Tachycardia (number)	69	8	0.858	0.692
Syncope (number)	2	13	1.04	0.96
Age (mean, SD)	65, 16.11	57.6, 18.5	1.024	0.006*
Chest pain (number)	14	158	0.464	0.018*

On multivariate logistic regression analysis (Table 3), increasing age showed a borderline significant positive association (OR: 1.018, p=0.055). Unilateral LL swelling/DVT demonstrated a significant positive correlation with PE, increasing the likelihood by 5.474 times (p=0.003). A significant negative association was observed for females, who were 0.308 times as likely as males to develop PE (p<0.001).

**Table 3:** Multivariate logistic regression analysis of PE risk factors * p<0.05 OR: Odds ratio, CI: Confidence interval, SOB: Shortness of breath, LL: Lower limb, DVT: Deep vein thrombosis, PE: Pulmonary embolism

Variables	OR	p-value	95% CI lower	95% CI upper
SOB	0.763	0.428	0.390	1.491
Tachycardia	0.847	0.708	0.355	2.019
Hypoxia	1.235	0.633	0.520	2.934
Haemoptysis	2.133	0.221	0.634	7.182
Unilateral LL swelling/DVT	5.474	0.003*	1.776	16.870
Syncope	0.614	0.573	0.112	3.348
Chest pain	0.604	0.218	0.270	1.348
Pleuritic chest pain	1.299	0.577	0.518	3.257
Age	1.018	0.055	1.000	1.037
Female	0.308	<0.001*	0.161	0.588
D-dimer	1.185	0.623	0.601	2.337
Procoagulation conditions	1.114	0.928	0.105	11.786
Previous DVT/PE	2.855	0.101	0.816	9.985
Malignancy	1.536	0.483	0.463	5.095
Immobilisation/long travel	2.590	0.174	0.656	10.225

Negative ORs for SOB, tachycardia, syncope, and chest pain suggest a lower likelihood of a PE diagnosis. Likewise, variables with positive odds, such as hypoxia, hemoptysis, and others, indicate a higher likelihood of a PE diagnosis. However, their associations were not statistically significant.

## Discussion

Among the risk factors studied, unilateral LL swelling/DVT emerged as a strong predictor for PE. They are well-known, documented risk factors for PE [2]. Interestingly, the female sex was associated with a reduced risk of PE. Though Lapostolle et al. [3] concluded that PE is increased among female long-haul travelers, de Miguel-Díez et al. [4] did not find a significant association between gender and the occurrence of PE. A 25-year population-based study in Minnesota [5] revealed a gender-specific pattern in venous thromboembolism incidence. Males had higher overall age-adjusted rates (1.2:1 male-to-female ratio), with a statistically significant difference. However, females showed higher rates during childbearing years, while males had higher rates after 45. This highlights the complexity of gender-specific patterns and warrants further investigation. However, it's important to consider that our study included more negative than positive CTPA and an unequal female-to-male ratio, which may have influenced our results.

The Mann-Whitney U test and univariate logistic regression revealed a significant association between age and the risk of PE. The analysis indicates that the risk of PE increases with age. However, when considering confounding factors such as immobilization and female sex in the multivariate logistic regression analysis, the relationship between age and PE risk was attenuated. Nevertheless, increasing age has been traditionally considered a risk factor for PE [6,7], where the incidence rate rises exponentially [8].

In our research, SOB and tachycardia were the two primary causes for conducting a CTPA. However, the multivariate logistic regression analysis revealed that both led to a decreased likelihood of PE. Despite being traditionally known clinical features of PE [7], the above finding could be due to the likely overutilization of CTPA (see below) and confounding factors. However, West et al.’s meta-analysis highlights the importance of sudden-onset SOB as a significant factor rather than just "any SOB" for PE [9]. In our study, however, the SOB was not explicitly characterized, which may have contributed to the observed results.

It is worth noting that while chest pain (without any characterization, OR: 0.604) was associated with lower odds of PE, pleuritic chest pain had higher odds (OR: 1.299). This discrepancy may be attributed to the more specific nature of pleuritic chest pain, which is characterized by a sharp, stabbing pain that worsens with deep breathing or coughing and is more indicative of pulmonary conditions, including PE [10]. Cloutier reported that the prevalence of pleuritic chest pain was 66% to 74%, while non-pleuritic chest pain was merely 14% among PE patients [7].

In the single-variable analysis, syncope was found to have a slightly positive but statistically insignificant relationship with PE (OR: 1.04, p=0.96). However, when we included multiple variables in the analysis, syncope appeared to have a weak negative and insignificant relationship with PE (OR: 0.614, p=0.573). Since no confounders were identified in our sensitivity analysis, the inconsistency between these results could be due to factors like suppressor variables, overfitting, sample size and statistical power, or random variation and sample error. Syncope is a well-known clinical feature of PE, with a pooled likelihood ratio of 2.38 in the above-mentioned meta-analysis [9].

Meanwhile, hemoptysis, prior DVT/PE, malignancy, immobilization/long travel, hypoxia, D-dimer levels, and procoagulation conditions exhibited positive ORs; none showed a statistically significant association with PE risk. The first four variables are part of the well-known clinical prediction tool, the Wells' score [11]. Furthermore, Kline et al. reported that increased D-dimer ordering was associated with an increased PE yield rate [12]. It is worthwhile to note that D-dimer is a sensitive but not specific test of PE [10], with a negative predictive value of 99.6% in a study [13].

The percentage of patients with PE in this sample is 12.9%, similar to the yields of previous Australian studies [14,15]. It is generally considered that CTPA positivity of less than 10% denotes its overuse [16]. Clements et al. noted that the overuse of CTPA is likely due to defensive medicine and the pressure on the ED for "4-hour disposition" [14]. 

Although only 2.53% of CTPA request forms included the actual Wells' score, in the GVH electronic request process, a pop-up window appears to guide clinicians in calculating the Wells' score and pre-test probability to determine the appropriateness of CTPA. However, the calculated Wells' score does not automatically appear in the submitted CTPA request form; clinicians must manually enter it. Given that our CTPA positive rate is close to 10%, it remains unclear whether the Wells' score in our system impacted the clinicians' decision to request CTPA.

In our study, we examined the relationship between the timing of CTPA requests and the occurrence of PE. No significant association was found between the time of the CTPA request and the presence of PE. One study in Hong Kong shows that the likelihood of diagnosing PE does not significantly vary across different time periods during the day [17]. Thurlow et al.'s paper shows that CTPA requests from senior clinicians had a higher PE yield [18]. In our study, we did not analyze the seniority of the clinicians requesting the imaging test.

Several limitations should be considered in this study. The single-center, retrospective design may limit generalizability and introduce potential biases. The reliance on medical records could affect the accuracy of the results (particularly for hypoxia, tachycardia, and non-aged-adjusted D-dimer), and the relatively small sample size may limit the study's statistical power. Regarding D-dimer levels, it is noteworthy that 60.8% of the CTPA request forms did not provide any information on this parameter. The absence of such data does not necessarily imply a negative D-dimer result. Additionally, unmeasured or residual confounding factors could influence the observed relationships. These limitations should be acknowledged when interpreting our research findings and planning future research.

## Conclusions

Our research indicates a strong association between unilateral LL swelling or confirmed DVT and PE. In our sample, factors such as increasing age, hemoptysis, hypoxia, pleuritic chest pain, positive D-dimer, prior DVT/PE, malignancy, immobilization, or long travel, and procoagulant conditions demonstrated positive ORs, suggesting potential associations with PE. Conversely, traditional risk factors like SOB and syncope show a decreased likelihood of PE, possibly due to the overutilization of CTPA in our sample. Though our study showed a significant negative association between the female gender and PE, this result must be interpreted cautiously due to the limitations of our sample and the complex nature of gender-specific patterns in PE occurrence, as demonstrated in other studies. Finally, the time of the CTPA request does not have a significant association with PE occurrence.
